# ‘Don’t forget the mouth!’: a process evaluation of a public oral health project in community-dwelling frail older people

**DOI:** 10.1186/s12903-021-01884-7

**Published:** 2021-10-18

**Authors:** Bach Van Ho, Claar Debora van der Maarel-Wierink, Annemiek Rollman, Roxane Anthea Francesca Weijenberg, Frank Lobbezoo

**Affiliations:** 1 Department of Orofacial Pain and Dysfunction, Academic Centre for Dentistry Amsterdam (ACTA), University of Amsterdam and Vrije Universiteit Amsterdam, Gustav Mahlerlaan 3004, 1081 LA Amsterdam, The Netherlands; 2 Department of Oral Medicine, Academic Centre for Dentistry Amsterdam (ACTA), University of Amsterdam and Vrije Universiteit Amsterdam, Gustav Mahlerlaan 3004, 1081 LA Amsterdam, The Netherlands

**Keywords:** Older adults, Oral health intervention, Oral health care, Health care, Interprofessional collaboration, Education and training

## Abstract

**Background:**

Older people are encouraged to remain community dwelling, even when they become care-dependent. Not every dental practice is prepared or able to provide care to community-dwelling frail older people, while their ability to maintain oral health and to visit a dentist is decreasing, amongst others due to multiple chronic diseases and/or mobility problems. The public oral health project ‘Don’t forget the mouth! (DFTM!) aimed to improve the oral health of this population, by means of early recognition of decreased oral health as well as by establishing interprofessional care. A process evaluation was designed to scientifically evaluate the implementation of this project.

**Methods:**

The project was implemented in 14 towns in The Netherlands. In each town, health care professionals from a general practice, a dental practice, and a homecare organization participated. The process evaluation framework focused on fidelity, dose, adaptation, and reach. Each of the items were examined on levels of implementation: macro-level, meso-level, and micro-level. Mixed methods (*i.e.*, quantitative and qualitative methods) were used for data collection.

**Results:**

The experiences of 50 health care professionals were evaluated with questionnaires, 22 semi-structured interviews were conducted, and the oral health of 407 community-dwelling frail older people was assessed. On each level of implementation, oral health care was integrated in the daily routine. On macro-level, education was planned (dose, adaption), and dental practices organized home visits (adaption). On meso-level, health care professionals attended meetings of the project (fidelity), worked interprofessionally, and used a screening-referral tool of the project DFTM! in daily practice (dose, adaption, reach). On micro-level, the frail older people participated in the screening of oral health (fidelity, dose), had their daily oral hygiene care observed (adaption) and supported if necessary, and some had themselves referred to a dental practice (reach). The semi-structured interviews also showed that the project increased the oral health awareness amongst health care professionals.

**Conclusions:**

The project DFTM! was, in general, implemented and delivered as planned. Factors that contributed positively to the implementation were identified. With large-scale implementation, attention is needed regarding the poor accessibility of the oral health care professional, financial issues, and increased work pressure.

*Trial registration* The Netherlands Trial Register NTR6159, registration done on December 13th 2016. URL: https://www.trialregister.nl/trial/6028

**Supplementary Information:**

The online version contains supplementary material available at 10.1186/s12903-021-01884-7.

## Background

In western countries, people are encouraged to remain community dwelling as long as possible, even when they become care-dependent [1–3], under the assumption that this contributes to their wellbeing [2]. While community dwelling, the older population will be dependent on the professional oral health care of regular dental practices [3]. However, not every oral health care professional in the regular setting is prepared or able to provide care to community-dwelling frail older people [4].

While the ability of frail older people to maintain good daily oral hygiene self-care [5] and to visit a dentist is decreasing, amongst others caused by multiple chronic diseases and/or mobility problems [5, 6], the risk for oral health problems is increasing [5, 7]. In addition, frail older people who are newly admitted to nursing homes since care can no longer be provided at home, often have a poor oral health, indicating that the deterioration of oral health took place while they were still community dwelling [8].

The public oral health project ‘Don’t forget the mouth! (the project DFTM!)’ was an initiative of general and oral health care organizations. This project aimed to improve the oral health of community-dwelling frail older people in The Netherlands [9], by means of early recognition of decreased oral health status, decreased ability for daily oral hygiene self-care, and the presence of oral health complaints as well as by establishing the need for interprofessional care. An effectiveness study was designed to assess the outcome of the project focusing on a subsample of community-dwelling older people with dementia [9].

Research on oral health projects is mainly focused on their effectiveness [10–12]. On the other hand, a process evaluation gives insight in the implementation of a project, enables the interpretation of the outcomes of the project, and provides an understanding of the context in which the project was implemented [11, 13]. The context of an intervention can be assessed in terms of compliance (*i.e.,* fidelity), execution (*i.e.,* dose), application (*i.e.,* adaptation), and reception *(i.e.,* reach). Understanding the context yields recommendations for improvements of the intervention, thus enabling large-scale implementation.

The present study was designed as a process evaluation with the primary aims to scientifically evaluate the implementation of the project DFTM!, to interpret the outcomes, to understand the context, and to provide recommendations for large-scale implementation. The secondary aims were to identify factors for success and barriers to implementation, and to gain insight in the oral health of community-dwelling frail older people.

## Methods

The public oral health project DFTM! was an initiative of SBT (clinic for special care dentistry), IDé (foundation for innovation in dementia), KNMT (professional association for dentists), ACTA (Academic Centre for dentistry Amsterdam), and VU (Vrije Universiteit Amsterdam, department of neuropsychology). A project group coordinated the implementation by bringing together the different healthcare professionals (of the general practice, dental practice, and home care organization), organizing meetings and providing education- and patient information materials. A research team designed and carried out the process evaluation.

### Implementation of the project ‘Don’t forget the mouth!’

The project DFTM! was implemented by the project group in 14 towns (Additional file 1) distributed over The Netherlands between September 2016 and October 2017. In each town, health care professionals from a general practice, a dental practice, and a homecare organization participated. The health care professionals attended two national and two regional meetings. The national meetings in September 2016 and June 2017 were focused on education about, for example, how to provide daily oral hygiene care and oral health in relation to general health, and were given by a geriatric dentist, a dental hygienist, and a geriatrician. The regional meetings took place at the beginning of the project’s implementation in the participating town, and 6 months after the start of the implementation. These meetings were focused on the regional oral health care organization and on creating interprofessional collaborations, for example by using the oral health screening-referral tool developed for the project DFTM!.

#### Recruitment of the health care professionals

The project group recruited the health care professionals in each town. They included professionals based on the following criteria: being prepared to incorporate oral health care in their organization (*e.g.,* including oral health in their policies and procedures); being prepared to use validated frailty questionnaires; having affinity with older people; having an accessible facility for older and/or disabled people; and being prepared to screen the oral health care needs of the community-dwelling older people for whom they care. Among the validated frailty questionnaires, the project group considered the use of the comprehensive geriatric assessment (CGA) [14], the Identification of Seniors At Risk (ISAR) screening questionnaire [15], Tilburg Frailty Indicator (TFI) [16], and the Groningen Frailty Indicator (GFI) [17]. Moreover, health care professionals in the towns could only participate when professionals from all three organizations (*i.e.*, a general practice, a dental practice, and a homecare organization) participated. The researchers excluded health care professionals with a temporary position in the organization, informed the health care professionals about the study through information letters, and written informed consent was obtained [9]. The study was approved by the Medical Ethical Committee of the VU Medical Centre (METc VUmc, with file number: 2016.406).

#### Education

Theoretical education was focused on the topics ‘oral health in relation to general health’ and ‘professional oral health care for frail older people’. Practical education was given on the topics ‘providing daily oral hygiene care in general’ and ‘daily oral hygiene care for non-cooperative patients or clients’. Moreover, the health care professionals were trained to use the oral health screening-referral tool, which was developed for the project DFTM! and which is further explained under the data collection section. Furthermore, district nurses were asked to give a practical course about daily oral hygiene care and oral health to their team of home care workers. A teaching programme for this practical course was made available, including a PowerPoint with a quiz and assignments to explore how to provide oral care as a team.

Educational materials about daily oral hygiene care, oral health, and professional oral health care were developed for the participating health care professionals, informal caregivers, and all community-dwelling older people. These were, and still are, available for everyone online (www.demondnietvergeten.nl and www.mondzorgbijouderen.info). Among these materials, there are six instructional videos about the following topics: the importance of a healthy mouth in older people, how to recognise problems in the mouth, brushing dentures, brushing natural teeth, how to give support in daily oral hygiene care, and oral care for non-cooperative patients or clients. Other components are the ‘Brush book’ (*i.e.,* ‘Poetsboek’), and materials and tools needed to give the practical course to home care workers.

### Process evaluation

The process evaluation framework to assess the context in which the project DFTM! was implemented focused on four items: fidelity, dose, adaptation, and reach. *Fidelity* is defined as the extent in which the project was implemented as intended (*i.e.,* quality of implementation and compliance) [11]. *Dose* is defined as the extent in which the project was offered and the amount of engagement to the project [11]. *Adaptation* is defined as the fitting of the method of the project into practice [11]. *Reach* is defined as the extent to which the project was delivered [11]. For an overview of the four items, see Table 1.

**Table 1:** Evaluation method of the project ‘Don’t Forget The Mouth!’ (DFTM!)

Characteristic	Defined as	Level of implementation	Tools	Aspect
Fidelity	Extent to which the project DFTM! was implemented as intended	Macro	Semi-structured interviews	Inclusion of oral health care Inclusion of screening-referral tool in the organization of the care
		Meso	Questionnaires	Attendance of the health care professionals to national and regional meetings
		Micro	Semi-structured interviews	The community-dwelling frail older people participate in the screening of the oral health (*i.e.,* prepared to let the oral health been screened)
Dose (delivered)	Extent the project DFTM! was offered	Macro	Questionnaires Semi-structured interviews	Planning the practical oral health care course Planning the project DFTM!
		Meso	Questionnaires Screening-referral tool	Frequency of use of the screening-referral tool
Dose (received)	The amount of engagement to the project DFTM!	Micro	Screening-referral tool	The community-dwelling frail older people actively participate in the screening of the oral health (*i.e.,* the screening-referral tool is filled out)
Adaptation	The fitting of the method of the project DFTM! into practice	Macro	Questionnaires	Organizing dental home visits Organizing the practical oral health care course
		Meso	Questionnaires and semi-structured interviews	Collaborations with health care professionals in a different discipline
		Micro	Screening-referral tool	Daily oral hygiene care observation
Reach	The extent to which the project DFTM! was delivered	Macro	Questionnaires	Giving the practical oral health care course to home care workers
		Meso	Questionnaires and semi-structured interviews	Interprofessional collaborations with health care professionals in a different discipline Daily oral hygiene care assistance
		Micro	Screening-referral tool and semi-structured interviews	Daily oral hygiene care Referral to an oral health care professional

Each of the four items were examined on three levels [18] of implementation: the organization (macro-level), the health care professionals (meso-level), and the community-dwelling frail older people (micro-level) [18] (Table 1). Mixed methods (*i.e.,* quantitative and qualitative methods [19]) were used for data collection [11, 13].

### Data collection

**Fig. 1:**
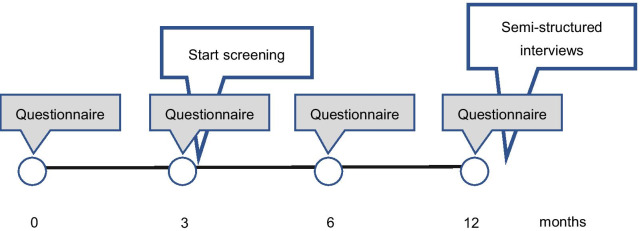
Timeline of the data collection in the process evaluation of the project DFTM!

At baseline and after 3, 6, and 12 months, the health care professionals completed questionnaires (viz., one for the general practice, one for the dental practice, and one for the home care organization) regarding interprofessional care and education. Furthermore, they were asked to use the screening-referral tool (see below) to examine the oral health status, daily oral hygiene self-care, and possible oral health complaints as well as need for interprofessional care of the community-dwelling frail older people for whom they care for and to share the anonymised data with the researchers. After 12 months, semi-structured interview appointments were made with the health care professionals, and interviews were conducted face-to-face at private locations (most frequently at the health care professionals’ practices) between March and May 2018 (Fig. 1). The different parts of the data collection are explained below.

#### Questionnaire

The researchers composed questionnaires for this study. There were three versions of the self-administered questionnaire, one suited for each type of care organization: the general practice, the dental practice, and the home care organization. The questionnaire contained open-ended questions regarding the same subjects: collaborations with other health care professionals, oral health care education, and organizing home visits for care and dental services.

#### Screening-referral tool

The oral health screening-referral tool was developed in Dutch specifically for the project DFTM! (Additional file 2) to support health care professionals to integrate oral health in their daily practice. The tool contains four parts with questions about the oral health status, oral health complaints, the last visit to a dental practice, and points to observe the older individual’s independence during daily oral hygiene care. The screening-referral tool can indicate whether the person needs support with the daily oral hygiene care, and/or whether referral to an (oral) health care professional is indicated. The tool itself can be used as a referral form. Referral is based on the preference of the older person, to either their dental practice or to the participating dental practice in the project DFTM!. If the older person does not have a dental practice anymore, referral is made to the participating dental practice. When referral is indicated but declined by the older person and/or his/her legal representative, the reason for declining is documented. For the process evaluation, the frequency in the use of the tool, the execution of the daily oral hygiene care observation, and the indication of the tool were assessed (Table 1). Furthermore, the screening-referral tool gives insight in the oral health of community-dwelling frail older people in the 14 towns.

#### Semi-structured interviews

The health care professionals in a participating town were approached for semi-structured interviews [20] and asked to share their experiences with the project DFTM!. These interviews were all conducted by the first author, recorded with a digital voice recorder (VN-4100PC, Olympus, Tokyo, Japan), and transcribed verbatim. Questions regarding the fidelity, dose, adaptation, and reach of the project DFTM! were determined by an expert panel, consisting of two members of the project group and two researchers (Additional file 3). During the interviews, the participants were encouraged to introduce other relevant topics. Besides the assessment of implementation of the project DFTM!, the semi-structured interviews also gave insight into factors for success and barriers to implementation.

### Data analysis

#### Quantitative analysis

The data of the screening-referral tool were entered in electronic case report forms built in Castor EDC (Castor electronic data capture, Ciwit BV, Amsterdam, The Netherlands). The demographics and the data of the screening-referral tool were presented with descriptive statistics, using IBM Statistics SPSS 25 (SPSS Inc., Chicago, IL, USA).

#### Qualitative analysis

The questionnaires were analysed by the first author according to the non-cross-sectional analysis [21, 22], using Microsoft Excel 10 (Microsoft, Redmond, WA, USA). Analysis was completed when the first two authors reached consensus on the conclusions. The semi-structured interviews were analysed according to the thematic analysis to identify patterns and recurrent themes derived from the data using a framework approach [22]. Each transcript was individually read by the first three authors to identify initial themes and subthemes. When saturation (*i.e.,* no new themes arose from the transcripts) was confirmed, the first author labelled the data by hand, and sorted the text in a thematic framework, created in Microsoft Excel 2010. Furthermore, the labelled text (*i.e.,* quotations) was assessed on positive, negative, or neutral contributions to implementation of the project DFTM!. Analysis was considered complete when the three authors refined the themes and reached consensus.

## Results

The results will be described first with numbers of recruited participants (health care professionals) and numbers of interviewed health care professionals, secondary with the results of the process evaluation assessed with the terms fidelity, dose, adaption and reach on macro-, meso, and microlevel, thirdly on factors of success and barriers to implementation, and fourthly with characteristics of the oral health of the community- dwelling older people.

### Participants

The project group DFTM! invited professionals from 17 towns to participate in the project DFTM!. In 14 towns, a triad of health care professionals accepted the invitation, resulting in fifty-eight health care professionals participating in the project DFTM!. All of them were also invited to participate in this study. Eight of them were excluded from this study because of a temporary position in the organization, because other health care professionals of the town stopped participating in the project before this study started, or because of absence of informed consent. Hence, a total of 50 health care professionals participated in this study at baseline, as shown in Table 2a.

**Table 2:** Characteristics of the health care professionals participating in the questionnaires (a) (N = 50), and the health care professionals participating in the interviews (b) (N = 22)

		N (%)
*(a)*		
Gender	Male	9 (18.0)
	Female	41 (82.0)
General practice	Physician	6 (12.0)
	General practice nurse	5 (10.0)
	Nurse	3 (6.0)
Dental practice	Dentist	13 (26.0)
	Dental hygienist	3 (6.0)
	Dental assistant	2 (4.0
Home care organization	District nurse	17 (34.0)
	Home care worker	1 (2.0)

		N (%)	N missing (%)
*(b)*			
Gender	Male	3 (13.6)	
	Female	19 (86.4)	
Age, mean (range, SD)		47.0 (27–62, 11.4)	1 (4.5)
Practice	Solo	1 (4.5)	
	Group	18 (81.8)	
	Both	3 (13.6)	
General practice	Physician	2	
	General practice nurse	2	
	Nurse	2	
Home care organization	District nurse	8	
	Home care worker	0	
	Other	1	
Dental practice	Dentist	5	
	Dental hygienist		
	Dental assistant	2	

*SD* standard deviation

Twenty-two health care professionals were interviewed (Table 2b). They were experienced as well as new graduates. The health care professionals brought up an additional topic during the semi-structured interviews, viz., ‘financial issues’. This topic was added after the fourth interview (Additional file 3). Saturation was reached when the health care professionals of three towns were interviewed. To confirm saturation, the health care professionals of the largest town and one of the smallest towns were also invited for an interview to identify if there were any themes left out in relation with the size of a town. This was not the case. The duration of a semi-structured interview ranged from 15 to 56 min, with a mean duration of 31 min. One semi-structured interview was done with two district nurses at the same time. Another short interview about the oral health education in the dental practice was performed with a dental assistant based on the recommendation of the dentist.

### Process evaluation

#### Fidelity

The project implementation was assessed for compliance on macro-level (organization) with semi-structured interviews, on meso-level (health care professionals) with questionnaires, and on micro-level (older people) with semi-structured interviews. Oral health care and the screening-referral tool were integrated in the health care organizations and the daily routine of the health care professionals. The opinions on using the screening-referral tool were divided, on the one hand it was difficult and not feasible to use, on the other hand it was easy and feasible to use. From the interviews, it became clear that this division is related to the phase of implementation. A district nurse described: *‘In the beginning, they said: here is another list to fill in, and we already have so many. Everything is done with lists. But we did it (…) Actually, it was only five minutes of work with the clients.*’ (4.3.111).

Overall, the screening-referral tool was integrated in the regular care. Furthermore, more health care professionals attended the regional meetings compared to the national meetings. The health care professionals of general practices participated less in the national meetings, with the required time investment as the main reason to not attend. A physician wrote down in the questionnaire: *‘No time or priority.’* (HP08.4). A general practice nurse added: *‘Choices have to be made with regard to education.’* (HP07.4).

The health care professionals considered the meetings as an added value for the implementation. A considerable number of community-dwelling frail older people were prepared to let the health care professionals screen their oral health (N = 407).

#### Dose

The project implementation was assessed for execution on macro-level (organization) with questionnaires and semi-structured interviews, on meso-level (health care professionals) with questionnaires and the screening-referral tool, and on micro-level (the older people) also with the screening-referral tool. The practical oral health care courses were planned by the district nurses. After 12 months, only the district nurse of one town was still planning to organize the course. Furthermore, the work pressure was raised for all health care professionals with implementing the project DFTM!, due to the time investment it required. Nevertheless, the health care professionals frequently used the screening-referral tool and shared the results (N = 407) with the researchers (Table 3). The health care professionals working at a home care organization performed 79.1% of the screenings (Table 3). In general, the health care professionals filled out the screening-referral forms completely (Tables 4, 5). The professionals of the general practice and dental practice filled out a small amount of screening-referral tools, viz., 6.1% and 7.1%, respectively. A general practice nurse said: *‘Only if people say that there are oral problems, I use the screening-referral tool.’* (1.3.16). Another general practice nurse said: *‘I would like to keep using the screening-referral form (…). It is meaningful, especially in diabetic care.’* (2.3.49). A dentist said: *‘I usually get the screening-referral form as referral, but I do not use it myself yet.’* (2.4.60).

**Table 3:** Screening-referral tool forms completed by health care professionals

Part of The Netherlands	N (towns)	Organization	N (%)	Total N (%)
North	7	Home care organization	162 (39.8)	211 (51.8)
		Dental practice	19 (4.7)	
		General practice	11 (2.7)	
		Unknown	19 (4.7)	
Middle	5	Home care organization	80 (19.7)	110 (27.1)
		Dental practice	10 (2.5)	
		General practice	11 (2.7)	
		Unknown	9 (2.2)	
South	2	Home care organization	80 (19.7)	83 (20.4)
		Dental practice	0 (0.0)	
		General practice	3 (0.7)	
Unknown		Unknown	3 (0.7)	3 (0.7)
Total	14	Home care organization	322 (79.1)	407 (100.0)
		Dental practice	29 (7.1)	
		General practice	25 (6.1)	
		Unknown	31 (7.6)

**Table 4:** Results of the screening-referral tool with community-dwelling frail older people (N = 407)

		N (%)	N missing (%)
Natural dentition	Yes	161 (39.6)	15 (3.7)
	No	231 (56.8)	
Dental prosthesis	Yes	268 (65.8)	33 (8.1)
	No	104 (25.6)	
	Unknown	2 (0.5)	
	Complete dental prostheses	189 (46.4)	28 (6.9)
	Maxillary	47 (11.5)	
	Mandibular	4 (1.0)	
Partial dental prosthesis	Yes	79 (19.4)	81(19.9)
	No	243 (59.7)	
	Unknown	7 (1.0)	
	Maxillary	51 (12.5)	
	Mandibular	46 (11.3)	
Retained roots	Yes	33 (8.1)	42 (10.3)
	No	264 (64.9)	
	Unknown	68 (16.7)	
Dental implants	Yes	39 (9.6)	32 (7.9)
	No	325 (79.9)	
	Unknown	11 (2.7)	
Time since last consultation with a dental practice?	< 1 year	145 (35.6)	12 (2.9)
	1–2 year	47 (11.5)	
	2–5 year	52 (12.8)	
	> 5 year	104 (25.6)	
	Unknown	47 (11.5)	
Do you have complaints in your mouth, in area surrounding your dental prosthesis, or of your teeth/molars?	Yes	72 (17.7)	17 (4.2)
	No	310 (79.5)	
	Unknown	8 (2.0)	
Can you chew well?	Yes	344 (84.5)	14 (3.4)
	No	46 (11.3)	
	Unknown	3 (0.7)	
Do you wear your dental prosthesis?	Yes	299 (73.5)	40 (9.8)
	No	65 (16.0)	
	Unknown	3 (0.7)	
Is your dental prosthesis loose?	Yes	99 (24.3)	42 (10.3)
	No	259 (63.6)	
	Unknown	7 (1.7)	
Do you have a dry mouth?	Yes	128 (31.4)	14 (3.4)
	No	254 (62.4)	
	Unknown	14 (3.4)	
Do you have a bad breath?	Yes	36 (8.8)	19 (4.7)
	No	330 (81.1)	
	Unknown	22 (5.4)

**Table 5:** Observation of the daily oral hygiene self-care of the community-dwelling frail older people (N = 407)

		N (%)	N missing (%)
Does the client brush on his/her own initiative in the morning and evening?	Yes	286 (70.3)	50 (12.3)
	No	61 (15.0)	
	Unknown	10 (2.5)	
Does the client brush on request?	Yes	210 (51.6)	79 (19.4)
	No	87 (21.4)	
	Unknown	31 (7.6)	
Does the client make effective brush movements?	Yes	241 (59.2)	66 (16.2)
	No	37 (9.1)	
	Unknown	63 (15.5)	
Does the client brush the teeth/molars and/or dental prosthesis on three sides (inside, outside and upside)?	Yes	263 (64.6)	65 (16.0)
	No	39 (9.6)	
	Unknown	40 (9.8)	
Does he/she carry on brushing at least one minute?	Yes	267 (65.6)	59 (14.5)
	No	42 (10.3)	
	Unknown	39 (9.6)	
Can the client rinse the mouth?	Yes	341 (83.8)	53 (13.0)
	No	7 (1.7)	
	Unknown	6 (1.5)	
Support is needed with:	Monitoring oral hygiene care	35 (8.6)	
	Assistance with, or taking over, oral hygiene care	20 (4.9)	
Is there an informal caregiver who could provide the correct support with daily oral hygiene care?	Yes	73 (17.9)	227 (55.8)
	No	107 (26.3)	

#### Adaptation

The project implementation was assessed for application on macro-level (organization) with questionnaires, on meso-level (health care professionals) with questionnaires and semi-structured interviews, and on micro-level (the older people) with the screening-referral tool. The majority of the dentists indicated that they would be willing to make a home visit, especially when the patient has mobility problems. A dentist wrote down on the questionnaire: *‘Yes, I make dental home visits to make dentistry accessible.’* (TP10.2). Another dentist added: *‘Especially for older people with mobility problems. Will outpatient dental services be the future?’* (TP06.2). Some dentists, however, raised questions about organizing dental home visits regarding infection prevention and lack of financial compensation.

Furthermore, the health care professionals experienced positive conversations with professionals in other disciplines. The initial contact between the health care professionals was made on the regional meetings in their own town, forming short communication lines.

The health care professionals did not all observe daily oral hygiene care with equal frequency. The results of the daily oral hygiene care observation on the screening-referral tool form showed some missing data, viz., in 12.3–19.4% of the cases (Table 5). In the questionnaire, a district nurse described the difficulty with a daily oral hygiene care observation: ‘*Observation of self-care is difficult. The client often has his/her own habits for daily oral hygiene care. They perform their oral hygiene care on moments that we are not there.*’ (TZ04.3). Another district nurse described the added value of the observation:

*There were people who showed their daily oral hygiene care perfectly, especially the older generation that could do the self-care themselves (…). A large group of people with dementia did an attempt, but you could see them brush for a short time only. They rinsed the mouth very quickly.’* (4.3.112).

#### Reach

The project implementation was assessed for reception on macro-level (organization) with questionnaires, on meso-level (health care professionals) with questionnaires and semi-structured interviews, and on micro-level (the older people) with the screening-referral tool and semi-structured interviews. The practical oral health care courses were given by the district nurses in collaboration with the dentists. A district nurse wrote down in the questionnaire: ‘*The course was given by the district nurse in collaboration with the dentist in the team*.’ (TZ04.3). A dentist added: ‘*The practical course was nice to give. In particular giving practical input.*’ (TP26.4).

Furthermore, the health care professionals experienced positive collaborations with professionals in other disciplines, and these collaborations continued after the initial meetings. A district nurse wrote down on the questionnaire: ‘*We have contact with the physicians and dental professionals from the project. Three or four times a year*.’ (TZ16.4).

A general practice nurse wrote: *‘Every six weeks, we have consultations with the home care professionals, and every six months the dentist is included.’* (HP10.4).

After 3 months from baseline, there were consultations between health care professionals of different disciplines. After 6 months, the frequency of these consultations decreased, while after 12 months the frequency increased again. Moreover, the health care professionals described in the semi-structured interviews that the project DFTM! improved the awareness of the importance of oral health and the identification of oral health problems in community-dwelling frail older people with the screening-referral tool.

When referral of a community-dwelling older person to a dental practice was needed, 19.9% of the people agreed with referral while 64.9% did not. Different reasons were given to decline referral, such as thinking it is not important. Familiarity with another dental practice, and the preference to make his or her own appointment meant that an appointment could not be made immediately which caused (undesirable) delays. In the semi-structured interviews, health care professionals indicated that the bond of trust of the older people in their health care professional contributed positively to the conversation, not only about the dental visits, but also about the daily oral hygiene care. Furthermore, the health care professionals observed (Table 5) with the screening- referral tool that 15.0% of the screened community-dwelling older people did not initiate daily oral hygiene care themselves, 21.0% did not even brush their teeth on request of the professional, and 10.3% could not brush for at least one minute.

### Factors for success and barriers to implementation

The thematic analysis of the semi-structured interviews resulted in six themes: collaboration, oral health awareness, work pressure, accessibility of the oral health care professional, financial issues, and work procedure. Oral health awareness is a result of the project DFTM!; the others themes described the factors for success and barriers, which are described with subthemes in Table 6. The six themes are explained one by one.

**Table 6:** Framework of the semi-structured interviews

Theme		Subtheme	Positive	Negative	Neutral	Subtheme quotes total	Theme quotes total
Collaboration	1.1	Health care professionals come into contact with each other, forming short communication lines, and strengthening the collaborations	135	32	22	189	
	1.2	Signalling oral health status and problems	56	5	8	69	
	1.3	Incorporating the implementation project DFTM! in existing structures (e.g. diabetic care)	33	10	10	53	
	1.4	Informal caregiver	13	4	4	21	
	1.5	The leader for the collaboration	4	1	10	15	
	1.6	Electronic Patient File	4	6	3	13	
	1.7	Organizing a home visit	10	0	0	10	
	1.8	Responsibility of the dentist to not lose sight of their patients	0	4	3	7	
							377
Oral health awareness	2.1	Awareness of the importance of oral health	97	5	7	109	
	2.2	Start the conversation about daily oral hygiene care and dental visits	32	8	2	42	
	2.3	Interaction between oral health and general health	28	3	3	34	
	2.4	National attention	3	4	17	24	
	2.5	Dealing with resistance during daily oral hygiene care	16	1	4	21	
	2.6	Daily oral hygiene care	12	4	1	17	
	2.7	Home visits of a dentist may reduce the threshold for older people	15	0	1	16	
	2.8	Enthusiasm of older people	11	0	2	13	
	2.9	Oral health is an overlooked subject	12	0	0	12	
	2.10	The bond of trust between the home care organization contributes to awareness	8	0	1	9	
	2.11	Carers experience the mouth as dirty	1	2	2	5	
	2.12	Other subthemes				10	
							312
Work procedure	3.1	Materials	21	14	12	47	
	3.2	Tools	8	12	9	29	
	3.3	Education	17	4	3	24	
	3.4	Other subthemes				18	
							118
Accessibility	4.1	Accessibility of the oral health care professional	25	25	9	59	
	4.2	Dependence on an informal care giver	1	6	0	7	
	4.3	Accessibility of the dental practice concerning the building	4	1	0	5	
	4.4	Accessibility of the dental practice concerning the journey	1	2	0	3	
							74
Financial issues	5.1	Dental care costs of older people	3	25	5	33	
	5.2	Reimbursement of home visit is insufficient for the dentist	8	1	3	12	
	5.3	The full care costs of older people	1	7	2	10	
	5.4	No financial threshold	1	1	0	2	
							57
Work pressure	6.1	Work pressure (*i.e.,* administrative tasks, time) of health care professionals	4	23	2	29	
	6.2	Designated time for the health care professional to spend on education to roll out the implementation project	3	0	0	3	
							32
Other issues		Other subthemes				17	17
Total quotes			587	210	145	987	987

#### Collaboration

It was found that a signalling role of district nurses, home care workers, and the general practice nurses was of great added value for interprofessional collaborations. A dentist said:

*‘This is a unique way to approach the older people through the home care organization. There could be added value if they not only do wound care, but could also play a signalling role in oral health care; that could be very positive.’* (5.3.139).

Communication and reporting back to each other were indicated as important. A factor for success of the project DFTM! was also the use of existing care networks, *e.g.,* networks for diabetic care and chronic obstructive pulmonary disease. A nurse said: *‘With diabetic care, there are structured visits (…) with test results and even more questionnaires. So, oral health became part of the conversation.’* (4.4.118). A general practice nurse added: *‘Oral health could also be discussed with chronic obstructive pulmonary disease patients with pneumonia.’* (2.3.53).

It was also mentioned that the dentist should not lose sight of the frail older people in his/her practice, and the added value of home visits organized with other health care professionals was stressed. Moreover, it was important to involve the informal caregiver, both for performing daily oral hygiene care as well as for organizing a visit to the dental practice. An important conclusion was also that in each town, one health care professional should be in charge of coordination to ensure the continuity of interprofessional collaboration.

#### Oral health awareness

Implementation of the work procedure of the project DFTM! contributed to awareness of the importance of oral health, and, on top of that, awareness of the relationship between oral health and general health in community-dwelling older people. Health care professionals of the home care organization and general practice nurses were aware that oral health is an overlooked subject. A district nurse said: *‘I think it’s a great idea, because I notice that barely any teeth are brushed in the neighbourhood, by the home care organization (…). Oral health care is really an overlooked subject.’* (4.2.100).

Sometimes, health care professionals experienced resistance during daily oral hygiene care, particularly with community-dwelling older people with cognitive problems. Recognising intimacy and autonomy, but also discussions with colleagues and looking for solutions like a dental home visit were mentioned and were part of the implementation. A district nurse said:

*‘They could always do everything themselves, it is a very intimate part. So they would like to do that themselves. Than you try different approaches (…) in order to maintain the client's sense of autonomy.’* (3.3.86-7).

Furthermore, broadening the awareness of the importance of oral health of community-dwelling older people to their informal caregivers and health care professionals, with nation-wide attention, was found of importance.

#### Work procedure

Both the regional meetings and the national meetings contributed to the implementation. A general practice nurse described in the semi-structured interview: *‘I liked the national meetings and found the subject of oral side effects of medication an eye-opener. I had no idea that there were so many side effects.’ (2.3.51).*

#### Accessibility of the oral health care professional

The accessibility of dental practices was mentioned both positively and negatively. Positive factors were the willingness of the oral health care professional to make home visits, to collaborate with the home care organization, and to think along with the community-dwelling older people within their possibilities and impossibilities. Negative factors mentioned were fear and a bad image of the dentist. A dentist said: *‘The accessibility of dental practices leaves much to be desired. People experience a barrier to go to the dentist. (…) People are afraid of criticism and are also afraid to go for treatment.’* (4.1.98).

Other barriers to visit a dental practice for community-dwelling frail older people were limited physical access to the practice, immobility of the person, and the dependence on informal care givers.

#### Financial issues

The general care costs, but especially the dental care costs were a barrier for the community-dwelling frail older people to make use of care facilities. A district nurse said:

*‘In this neighbourhood, a lot of people are struggling. And the dental costs are a big problem. They simply don’t go to the dentist to save money.’* (2.5.65).

Furthermore, it was noted that the reimbursement of home visits was insufficient for dentists.

#### Work pressure

Time designated for oral health care or education on this subject contributed positively to the implementation. A district nurse explained: *‘There is a lot to do. For example I make the bed, turn on the light, prepare a meal; a lot of things. You hope that everyone can brush their teeth for as long as possible.*’ (1.4.21). Another district nurse said: *‘So, when we make the general care plan as district nurse, we can designate time for everything that is needed. You can also designate time for oral health care (…); that isn’t a problem.’* (4.3.113).

In short, the oral health awareness of the health care professionals increased after participating in the project DFTM!. The factors contributing positively to implementation of the project (*i.e.,* success factors) were: the signalling role of district nurses, home care workers, and the general practice nurses, the use of existing care networks, the regional and national meetings, and time designated for oral health care and education. Barriers to implementation of the project were: the poor accessibility of the oral health care professional, financial issues, and increased work pressure.

### Oral health of community-dwelling older people

The 407 screened community-dwelling frail older people had a mean age of 84.7 (standard deviation = 7.6, range = 65–104,), and 67.4% was female. Table 4 shows the results of the screening-referral tool. Of all screened community-dwelling frail older people, 39.6% had (part of) their natural dentition, 8.1% showed retained roots, and 9.6% had dental implants. In the last year, 35.6% of this population visited a dental practice. Older people with a natural dentition visited an oral health care professional more often than older people with complete dental prostheses, 67.1% versus 14.5%. There were complaints in the mouth in the area surrounding the dental prosthesis or teeth/molars in 17.7% of this population, 24.3% had complaints of insufficient retention of their dental prostheses, and 31.4% had complaints of a dry mouth.

## Discussion

The primary aim of this study was to scientifically evaluate the implementation of the project ‘Don’t forget the mouth!’ (DFTM!); to interpret the outcomes, to understand the context, and to provide recommendations for large-scale implementation, with a process evaluation framework focused on the fidelity, dose, adaptation, and reach. These items were examined on three levels of implementation: the organization (macro-level), the health care professionals (meso-level), and the community-dwelling frail older people (micro-level). The secondary aims of this study were to identify factors for success and barriers to implementation, and to gain insight into the oral health of community-dwelling frail older people. On each level of implementation the oral health care was incorporated in the daily routine. On macro-level dental practices organized home visits and contributed to the practical oral health care courses (adaptation), while home care organizations planned and organized the practical oral health care courses (dose, adaption). On meso-level health care professionals attended to national and regional meetings (fidelity), worked interprofessionally, and used the screening-referral tool in daily practice (dose, adaption and reach). On micro-level, the community-dwelling frail older people participated in the screening of their oral health (fidelity, dose), had their daily oral hygiene care observed (adaption), and had themselves supported in their daily oral hygiene care if necessary, while some had themselves referred to an oral health care professional (reach). In a recent scoping review [23], a range of oral health care interventions were described. Many studies only focus on one care level (*i.e*., patient or carer), while in the current study we conducted a process evaluation on three levels. The factors contributing positively to implementation of the project (*i.e.,* success factors) were: the signalling role of district nurses, home care workers, and the general practice nurses, the use of existing care networks, the regional and national meetings, and time designated for oral health care and education. Barriers to implementation of the project were: the poor accessibility of the oral health care professional, financial issues, and increased work pressure. In the group of community-dwelling frail older people, 39.6% had (part of) their natural dentition, 9,6% had dental implants, in 17.7% there were complaints in the mouth in the area surrounding the dental prosthesis or teeth/molars, and 31.4% had complaints of a dry mouth.

The process evaluation of the project DFTM! showed that the *fidelity* is related to the phase of implementation, and that focusing on regional meetings could improve the compliance. In the semi-structured interviews, the participation of the community-dwelling frail older people in the screening of their oral health was insufficiently discussed. Nevertheless, a considerable number of individuals were prepared to let the health care professionals screen their oral health (N = 407). The increased work pressure had negative influence on the *dose*, and was also found a barrier for implementing the project. Time designated for oral health care or education on this topic contributed positively to the implementation of the project. The screening-referral tool was often used with the home care organization, but less at the general practice and the dental practice. Integrating questions from the tool into existing instruments or systems in the general practice could contribute to the use. For health care professionals in the dental practice, the added value of the screening-referral tool could be that it helps putting more emphasis on daily oral hygiene self-care and interprofessional collaboration with district nurses to improve the daily oral hygiene care. In the literature, there are alternative assessment tools available to monitor oral health, which have been tested for reliability and validity [24]. The Oral Health Assessment Tool and the Revised Oral Assessment Guide are the most relevant, because they are the most complete in assessing the overall oral health and have good measurement properties and methodology [24]. The screening-referral tool of DFTM! is unique and could contribute to the assessments in evaluation of the daily oral hygiene self-care, so that an older person can be supported in a timely manner. With fitting the project DFTM! into practice (*i.e.,* adaptation*),* the health care professionals experienced positive conversations with professionals of other disciplines, and formed short interprofessional communication lines. There are many oral health care projects implemented worldwide, and all focus on primary prevention [23]. The project DFTM! has many resemblances, but sets out to differentiate with the interdisciplinary collaborations on the local level. In contrast with a recent systematic review [25], the majority of the dentists in this study were willing to make home visits, especially when patients have mobility problems. In line with the systematic review [25], the dentists mentioned in the semi-structured interviews that home visits require more time. Guidelines and education in geriatric dentistry could be considered to act as facilitators to improve the oral health care for community-dwelling frail older people [25]. Furthermore, regarding *reach,* not only the work procedure of the project DFTM! was implemented, but also increased oral health awareness was achieved.

To broaden the awareness of the importance of oral health in community-dwelling older people themselves, their informal caregivers, and health care professionals, nation-wide attention might be a good start, as suggested in the semi-structured interviews as well as in a recent study [26].

Additionally, the current study indicated that district nurses, home care workers, and the general practice nurses could play a signalling role in the early identification of decreased ability for daily oral hygiene self-care and dental visits. An general practitioner could also fulfil this role. Subsequently, the district nurse can include assistance with daily oral hygiene care in the general health care plan, and assist with organizing a visit to the dental practice. In this way, deterioration in oral health can be prevented. As mentioned, district nurses and home care workers recognized oral health as an overlooked subject, but also the intimacy of assistance with daily oral hygiene care and the necessary respect for the autonomy of the frail older person were mentioned. Further, they discussed oral health care-related resistant behaviour with colleagues to find solutions. These findings are valuable parts of the project DFTM! and could contribute to self-efficacy of these professionals in providing assistance with daily oral hygiene care.

The mid-term sustainability of the DFTM! project was partly investigated with the semi-structured interviews conducted after 12 months of implementation. The results showed that after 12 months, the interprofessional collaboration and the awareness of the importance of oral health as well as the identification of oral health problems were maintained. Evaluation also showed that it is important for the long term that there is a leader in each town who takes the initiative in organizing interprofessional meetings, so that continuity is guaranteed. The leader can come from any discipline.The results of the screening-referral tool showed that 64.9% of the community-dwelling frail older persons declined a direct referral to an oral health care professional. One of the reasons was the assumption that oral health is not important. A qualitative study [5] showed that older people often lack the motivation to visit the dentist because of decrease in perceived importance due to deterioration of their general health. Other studies [27, 28] also showed decrease of dental service utilization, especially in edentulous older people. Another reason for declining referral, given in the semi-structured interviews by the health care professionals, was a financial issue. In The Netherlands, general dental services for adults are not covered by the standard health insurance [29]. This could contribute to this barrier [30]. Besides these barriers, the accessibility of the oral health care professional was also mentioned in the semi-structured interviews. In the end, a recent study concluded that underlying functional loss is the significant contributor in avoiding health care services [30].

Moreover, the results of the screening-referral tool showed various oral health problems in the community-dwelling frail older people, confirming the assumption that the deterioration in oral health begins when people become care dependent and still live at home [8].

Recently, collaborating researchers of The Netherlands and Flanders (Belgium) shared their research agenda focused on oral health and oral function of older people, interprofessional collaboration within primary care, and cost benefits and long-term effect(s) of sustainable oral health care throughout the total life course [31]. Further research and development of the project DFTM!, such as assessing the optimal role of each individual health care professional, and focusing on the oral health care needs of frail older people, might yield a valuable contribution to this research agenda.

### Strengths and limitations

This study has several strengths and weaknesses. A strong point of this study is the large scale of the process evaluation, which strongly contributes to the validity of the present outcomes. In addition, the participating health care professionals were prepared to include oral health care in their daily practice, which could be interpreted as motivation and could have influenced the results positively. Although this could be considered as selection bias, according to the Capability, Opportunity, Motivation, Behaviour (COM-B) model of behaviour change, used for designing interventions, the factor ‘motivation’ on its own cannot achieve change [32]. Furthermore, the results of the process evaluation of the DFTM! project are specific for the Dutch population and care system. However, the success factors and barriers are also of value for implementation of other public oral health projects for community dwelling older people. The screening-referral tool was frequently used and generally appreciated, but it is not yet validated. This could be considered as a weakness of the current study. It could be argued, however, that the instrument has sufficient face validity, *i.e.*, the tool can be subjectively viewed as covering the concepts it purports to measure [33]. Nevertheless, further validation of the tool will be the aim of a future study. In addition, a translation and cultural adaptation into English is planned as to enable its international roll-out.

### Suggestions for future research and implementation

The project DFTM! was scientifically evaluated at three implementation levels, of which the macro-level and micro-level could be further assessed in future research with semi-structured interviews with policymakers, managers of home care organizations, informal caregivers, and community-dwelling frail older people. The project DFTM! contributed to more awareness about the importance of oral health in community-dwelling older people and of interprofessional collaborations amongst the participating health care professionals. Additionally, interprofessional collaborations contributed positively to the accessibility of oral health care. Therefore, a nation-wide implementation is recommended after elaboration, taking into account the identified barriers. Furthermore, broadening national awareness of the importance of oral health in older people and stimulating health care professionals to organize care networks will strengthen the roll-out and feasibility of the project, and could contribute to the oral health and quality of life of community-dwelling older people. For daily practice, it is important to train young health care professionals in interprofessional care, with mutual learning between disciplines, and to establish interprofessional collaborations in the field, with the ultimate purpose to contribute to good (oral) health care.

## Conclusion

The project DFTM! was, in general, implemented and delivered as planned on macro-, meso-, and micro-level, and oral health care was integrated in the daily routine. The project DFTM! led to interprofessional collaborations between the general practices, dental practices, and home care organizations, whereby the topic of oral health has become part of the daily routine of the health care professionals, and contributed to awareness about the importance of oral health of community-dwelling frail older people. Factors contributing positively to implementation of the project were the signalling role of district nurses, home care workers, and general practice nurses, the use of existing care networks, the regional meetings, and the time designated for oral health and education. With large-scale implementation of the project attention is needed regarding the poor accessibility of the oral health care professional, financial issues, and increased work pressure.

## Supplementary Information

**Additional file 1. **General data of the towns.

**Additional file 2.** The screening-referral tool.

**Additional file 3.** Determined topics for semi-structured interviews.

## Data Availability

As written the availability of other data and materials in this study are available from the corresponding author on reasonable request.

## Abbreviations

DFTM!: Don’t forget the mouth!
METc VUmc: Medical Ethical Committee of the VU Medical Centre
