# Supplementary material for: ‘Don’t forget the mouth!’: a process evaluation of a public oral health project in community-dwelling frail older people
Source: BMC Oral Health. 2021 Oct 18;21:536. doi: 10.1186/s12903-021-01884-7 (PMC8522087; doi:10.1186/s12903-021-01884-7)
Supplement: Supplementary file 1 — Additional file 1. General data of the towns. [file 12903_2021_1884_MOESM1_ESM.pdf]

ACTA
ACADEMIC CENTRE FOR DENTISTRY AMSTERDAM

‘Don’t Forget The Mouth !’: a process evaluation of a public oral health project in community-dwelling frail older people

Bach Van Ho[1], Claar Debora van der Maarel-Wierink[2], Annemiek Rollman[1], Roxane Anthea Francesca Weijenberg[1], Frank Lobbezoo[1]

[1] Department of Orofacial Pain and Dysfunction, Academic Centre for Dentistry Amsterdam (ACTA), University of Amsterdam and Vrije Universiteit Amsterdam, Gustav Mahlerlaan 3004, 1081 LA Amsterdam, The Netherlands

[2] Department of Oral Medicine, Academic Centre for Dentistry Amsterdam (ACTA), University of Amsterdam and Vrije Universiteit Amsterdam, Gustav Mahlerlaan 3004, 1081 LA Amsterdam, The Netherlands

Corresponding author: Bach Van Ho

email address: b.v.ho@acta.nl

Additional file 1: General data of the towns

Additional file 1: General data of the towns

General data of the year 2017 of the towns participating in the project ‘Don’t forget the mouth!’ (DFTM!) from Statistics Netherlands[1]

**Part of The Netherlands**	**Town**	**Population (x1.000)**	**Education level**			**Low income (%)**
			**Primary (%)**	**Secondary (%)**	**Tertiary (%)**	
North	N1	108.4	27.7	39.8	30.1	7.8
	N2	89.3	23.1	32.3	44.6	5.9
	N3	844.9	22.2	27.0	48.8	13.7
	N4	67.6	28.0	42.0	28.0	9.0
	N5	88.9	25.8	33.3	40.9	8.0
	N6	15.8	33.3	41.7	25.0	4.6
	N7	23.7	33.3	38.9	22.2	4.9
Middle	M1	48.3	25.0	36.1	36.1	3.9
	M2	21.0	31.3	43.8	25.0	3.8
	M3	22.7	29.4	41.2	23.5	4.4
	M4	16.5	25.0	41.7	33.3	5.1
	M5	36.1	22.2	40.7	33.3	4.2
South	S1	152.4	27.4	35.0	36.8	8.3
	S2	93.3	33.8	39.4	25.4	9.0
Average		116.4	27.7	39.8	30.1	6.6
The Netherlands		17081.5	28.9	38.7	31.0	7.9

[1] Statistics Netherlands. Population dynamics; birth, death and migration per region. Available from: https://opendata.cbs.nl/statline/#/CBS/en/dataset/37259eng/table?ts=1632209741827
Statistics Netherlands. Laag en langdurig laag inkomen; huishoudenskenmerken, regio (indeling 2019). Available from: https://opendata.cbs.nl/statline/#/CBS/nl/dataset/84641NED/table?ts=1582915219533
Statistics Netherlands. Arbeidsdeelname; regionale indeling 2019. Available from: https://opendata.cbs.nl/statline/#/CBS/nl/dataset/84703NED/table?ts=1582896098175
