# Supplementary material for: ‘Don’t forget the mouth!’: a process evaluation of a public oral health project in community-dwelling frail older people
Source: BMC Oral Health. 2021 Oct 18;21:536. doi: 10.1186/s12903-021-01884-7 (PMC8522087; doi:10.1186/s12903-021-01884-7)
Supplement: Supplementary file 2 — Additional file 2. The screening-referral tool. [file 12903_2021_1884_MOESM2_ESM.pdf]

ACTA
ACADEMIC CENTRE FOR DENTISTRY AMSTERDAM

‘Don’t Forget The Mouth !’: a process evaluation of a public oral health project in community-dwelling frail older people

Bach Van Ho[1], Claar Debora van der Maarel-Wierink[2], Annemiek Rollman[1], Roxane Anthea Francesca Weijenberg[1], Frank Lobbezoo[1]

[1] Department of Orofacial Pain and Dysfunction, Academic Centre for Dentistry Amsterdam (ACTA), University of Amsterdam and Vrije Universiteit Amsterdam, Gustav Mahlerlaan 3004, 1081 LA Amsterdam, The Netherlands

[2] Department of Oral Medicine, Academic Centre for Dentistry Amsterdam (ACTA), University of Amsterdam and Vrije Universiteit Amsterdam, Gustav Mahlerlaan 3004, 1081 LA Amsterdam, The Netherlands

Corresponding author: Bach Van Ho

email address: b.v.ho@acta.nl

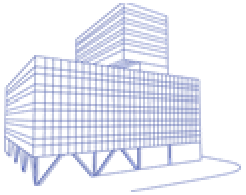

Additional file 2: The screening-referral tool developed by the project ‘Don’t forget the mouth!’

Additional file 2: The screening-referral tool

The screening-referral tool as developed by the project ‘Don’t forget the mouth!’

The original screening-referral tool is enclosed in Dutch, followed by an English translation.

De Mond niet Vergeten!

Screeningsformulier

Patiënt/Cliënt Naam: ○ M / V ○ geboortejaar:

Gebitssituatie

	ja/nee/onbekend
Hebt u eigen tanden en/of kiezen?	○ ○ ○
Hebt u een volledige gebitsprothese? Bij ja: ☐ boven ☐ onder	○ ○ ○
Hebt u een gedeeltelijke gebitsprothese (plaatje of frame)? Bij ja: ☐ boven ☐ onder	○ ○ ○
Hebt u nog eigen tandwortels onder uw gebitsprothese?	○ ○ ○
Hebt u implantaten?	○ ○ ○

Hebt u klachten in uw mond, rond uw gebitsprothese of aan uw tanden/kiezen?	○ ○ ○
Kunt u goed kauwen?	○ ○ ○
Draagt u uw gebitsprothese?	○ ○ ○
Zit de gebitsprothese los?	○ ○ ○
Hebt u een droge mond?	○ ○ ○
Hebt u een vieze mondgeur?	○ ○ ○

Bezoek aan de mondzorgpraktijk

Hoe lang geleden bezocht u voor het laatst een mondzorgpraktijk?

- ○ korter dan 1 jaar geleden
- ○ 1-2 jaar geleden
- ○ 2-5 jaar geleden
- ○ langer dan 5 jaar geleden
- ○ onbekend

Norm:
- *eigen tanden/kiezen: minimaal elk half jaar;*
- *volledige gebitsprothese: elk jaar.*

Mag ik u naar de mondzorgverlener verwijzen?

○ Ja, te weten naar:
- ○ seniorenproof praktijk
- ○ andere praktijk

naam:

○ Nee, reden:
- ☐ vind ik niet belangrijk
- ☐ angst voor de tandarts
- ☐ kosten
- ☐ anders, te weten:

Aandachtspunten voor de mondzorgverlener

- ☐ Extra aandacht nodig vanwege geheugen-aandoening
- ☐ Extra aandacht nodig vanwege lichamelijke aandoening
- ☐ Begeleiding door naaste bij de behandeling nodig: naaste contacteren (*zie Verwijsformulier*)
- ☐ Patiënt kan zich niet verplaatsten, ook niet per taxi: s.v.p. huisbezoek
- ☐ Anders, te weten:

Mondverzorging

Observatie zelfzorg

	ja/nee/onbekend
Poetst de cliënt op eigen initiatief 's ochtends en 's avonds?	○ ○ ○
Poetst de cliënt op verzoek?	○ ○ ○
Maakt de cliënt effectieve poetsbewegingen?	○ ○ ○
Poetst de cliënt de tanden/kiezen en/of de gebitsprothese aan de binnen-, buiten- en bovenkant?	○ ○ ○
Houdt hij/zij het poetsen tenminste een minuut vol?	○ ○ ○
Kan de cliënt de mond spoelen?	○ ○ ○

Conclusie

○ Zijn niet àlle vragen met 'ja' beantwoord? ➞ Hulp is nodig, te weten:
- ○ toezien op mondverzorging
- ○ assisteren of overnemen van de mondverzorging

○ Zijn alle bovenstaande vragen wel met 'ja' beantwoord? ➞ Geen hulp nodig

Als er hulp nodig is

	ja / nee
Is er een mantelzorger die de juiste hulp kan bieden bij de dagelijkse mondverzorging (conform het Poetsboek)?	○ ○

Hoe staat cliënt tegenover hulp bij de mondverzorging?

Additional file 2: The screening-referral tool developed by the project 'Don't forget the mouth!'

De Mond niet Vergeten!

Verwijsformulier

Gegevens patiënt / cliënt:

○ Dhr / Mw ○

Naam: ______________________

Adres: ______________________

Telefoon: ______________________

Geboortedatum: ______________________

BSN: ______________________

Moet iemand anders gebeld worden om een afspraak te maken?

○ Nee

○ Ja, te weten:

Naam: ______________________

Relatie tot patiënt/cliënt: ______________________

Telefoon: ______________________

Verwijzing: (toelichting in bijbehorend screeningsformulier)

☐ Verwijzing naar mondzorgpraktijk, naam:

☐ Verwijzing naar thuiszorg, naam: ______________________

Patiënt / cliënt (of diens wettelijk vertegenwoordiger) geeft toestemming voor deze verwijzing:

Plaats:

Datum: ______________________

Naam: ______________________

Relatie tot patiënt/cliënt: ______________________

Handtekening: ______________________

Verwijzer: Naam, functie en contactgegevens verwijzer (stempel mag ook)

Additional file 2: The screening-referral tool developed by the project ‘Don’t forget the mouth!’

Screening form

Patient/Client Name: O M / F O year of birth:

Oral health status

yes/no/unknown

Question	yes	no	unknown
Do you have a natural dentition?	O	O	O
Do you have a full dental prosthesis?	O	O	O
If Yes, o maxillary o mandibular	O	O	O
Do you have a partial dental prosthesis?	O	O	O
If Yes, o maxillary o mandibular	O	O	O
Do you have retained roots beneath your prosthesis?	O	O	O
Do you have dental implants?	O	O	O
Do you have complaints in your mouth, in area surrounding your dental prosthesis, or of your teeth/molars?	O	O	O
Can you chew well?	O	O	O
Do you wear your dental prosthesis?	O	O	O
Is your dental prosthesis loose?	O	O	O
Do you have a dry mouth?	O	O	O
Do you have a bad breath?	O	O	O

Visit to the dental practice

How long ago did you visit a dental practice?

O < 1 year
O 1-2 years
O 2-5 years
O >5 years
O Unknown

Norm:
- Natural dentition: minimally once every 6 months
- Complete dental prostheses: each year

May I refer you to an oral health care professional?

O Yes, to:
- O a senior-proof practice
- O another practice

Name:

O No, because:
- O I don't think it is important
- O fear of an oral health care professional
- O costs
- O other, namely:

Points of attention for the oral health care professional

O attention needed because of a cognitive decline
O attention needed because a physical decline
O informal caregiver needed for guidance during the treatment: contact with the informal caregiver (see Referral form)
O patient cannot be displaced, also not with a taxi service: please home visit
O other, namely:

Oral hygiene care

yes/no/unknown

Observation of daily oral hygiene self-care

Question	yes	no	unknown
Does the client brush on his/her own initiative in the morning and evening?	O	O	O
Does the client brush on request?	O	O	O
Does the client make effective brush movements?	O	O	O
Does the client brush the teeth/molars and/or dental prosthesis on three sides (inside, outside and upside)?	O	O	O
Does he/she carry on brushing at least one minute?	O	O	O
Can the client rinse the mouth?	O	O	O

Conclusion

O Not all the questions are answered with yes → Support is needed with:
- O Monitoring oral hygiene care
- O Assistance with, or taking over, oral hygiene care

O All questions above are answered with yes → no support is needed

If support is needed

Is there an informal caregiver who could provide the correct support with daily oral hygiene care?

O yes o no

What is the client's opinion about support in daily oral hygiene?

Additional file 2: The screening-referral tool developed by the project 'Don't forget the mouth!'

De Mond niet Vergeten!

Referral form

Data of the patient/client:

O Mr./Mrs. O
Name
Adress
Telephone
Birth date
Social Security Number

Does someone else need to be called for making an appointment?
O No
O Yes, namely :
Name:
Relation with patient/client:
Telephone

Referral (explanation corresponding to screening form)
O Referral to dental practice, name:
O Referral to home care organization, name:

Patient/ client (or their legal representative) gives permission for this referral:
Place
Date
Name
Relation with patient/client
Signature

Referrer:
Name, function and contact details referrer (stamp also valid)

Additional file 2: The screening-referral tool developed by the project 'Don't forget the mouth!'
