# Supplementary material for: ‘Don’t forget the mouth!’: a process evaluation of a public oral health project in community-dwelling frail older people
Source: BMC Oral Health. 2021 Oct 18;21:536. doi: 10.1186/s12903-021-01884-7 (PMC8522087; doi:10.1186/s12903-021-01884-7)
Supplement: Supplementary file 3 — Additional file 3. Determined topics for semi-structured interviews. [file 12903_2021_1884_MOESM3_ESM.pdf]

ACTA

ACADEMIC CENTRE FOR DENTISTRY AMSTERDAM

'Don't Forget The Mouth !': a process evaluation of a public oral health project in community-dwelling frail older people

Bach Van Ho[1], Claar Debora van der Maarel-Wierink[2], Annemiek Rollman[1], Roxane Anthea Francesca Weijenberg[1], Frank Lobbezoo[1]

[1] Department of Orofacial Pain and Dysfunction, Academic Centre for Dentistry Amsterdam (ACTA), University of Amsterdam and Vrije Universiteit Amsterdam, Gustav Mahlerlaan 3004, 1081 LA Amsterdam, The Netherlands

[2] Department of Oral Medicine, Academic Centre for Dentistry Amsterdam (ACTA), University of Amsterdam and Vrije Universiteit Amsterdam, Gustav Mahlerlaan 3004, 1081 LA Amsterdam, The Netherlands

Corresponding author: Bach Van Ho

email address: b.v.ho@acta.nl

Additional file 3: Determined topics for semi-structured interviews

Determined topics for semi-structured interviews about the implementation process and to identify factors for success and barriers of the project ‘Don’t forget the mouth!’ (DFTM!)

Topics	Sub topics
Implementation of the project DFTM!	
Expectations	
The population cared for by the health care professionals (factors for success and barriers)	
Collaborations (factors for success and barriers)	
DFTM! (factors for success and barriers)	Education<br>Website<br>Materials: screening-referral tool, brush book, leaflets, daily oral hygiene care products, and website
Outcome	Roadmap of the project DFTM!<br>Referral<br>Screening<br>Meeting (Multi-Disciplinary Meeting)
Describe situation	Intake<br>Daily oral hygiene care routine
Attitude towards the project DFTM!	
Future	
Additional subject	
Financial issues	
